# Liquid biopsy reveals collateral tissue damage in cancer

**DOI:** 10.1172/jci.insight.153559

**Published:** 2022-01-25

**Authors:** Asael Lubotzky, Hai Zemmour, Daniel Neiman, Marc Gotkine, Netanel Loyfer, Sheina Piyanzin, Bracha-Lea Ochana, Roni Lehmann-Werman, Daniel Cohen, Joshua Moss, Judith Magenheim, Maureen F. Loftus, Lauren Brais, Kimmie Ng, Raul Mostoslavsky, Brian M. Wolpin, Aviad Zick, Myriam Maoz, Albert Grinshpun, Anatoli Kustanovich, Chen Makranz, Jonathan E. Cohen, Tamar Peretz, Ayala Hubert, Mark Temper, Azzam Salah, Shani Avniel-Polak, Simona Grozinsky-Glasberg, Kirsty L. Spalding, Ariel Rokach, Tommy Kaplan, Benjamin Glaser, Ruth Shemer, Yuval Dor

**Affiliations:** 1 Department of Developmental Biology and Cancer Research, Institute for Medical Research Israel-Canada, The Hebrew University-Hadassah Medical School, Jerusalem, Israel.; 2 Neuropediatric Unit, Shaare Zedek Medical Center, Jerusalem, Israel.; 3 Department of Neurology, The Agnes-Ginges Center for Neurogenetics, The Hebrew University of Jerusalem, Jerusalem, Israel.; 4 School of Computer Science and Engineering, The Hebrew University of Jerusalem, Israel.; 5 Department of Medical Oncology, Dana-Farber Cancer Institute and Harvard Medical School, Boston, Massachusetts, USA.; 6 Massachusetts General Hospital Cancer Center, Harvard Medical School, Boston, Massachusetts, USA.; 7 Sharett Institute of Oncology, Hadassah Medical Center and the Faculty of Medicine, The Hebrew University of Jerusalem, Jerusalem, Israel.; 8 Department of Neurology and Oncology, Gaffin Center for Neuro-Oncology, Sharett Institute of Oncology, and; 9 Department of Endocrinology and Metabolism Service, Hadassah Medical Organization and The Faculty of Medicine, The Hebrew University of Jerusalem, Israel.; 10 Department of Cell and Molecular Biology, Karolinska Institutet, Stockholm, Sweden.; 11 Pulmonary Institute, Shaare Zedek Medical Center, Jerusalem, Israel.

**Keywords:** Cell Biology, Oncology, Epigenetics, Molecular diagnosis

## Abstract

Cancer inflicts damage to surrounding normal tissues, which can culminate in fatal organ failure. Here, we demonstrate that cell death in organs affected by cancer can be detected by tissue-specific methylation patterns of circulating cell-free DNA (cfDNA). We detected elevated levels of hepatocyte-derived cfDNA in the plasma of patients with liver metastases originating from different primary tumors, compared with cancer patients without liver metastases. In addition, patients with localized pancreatic or colon cancer showed elevated hepatocyte cfDNA, suggesting liver damage inflicted by micrometastatic disease, by primary pancreatic tumor pressing the bile duct, or by a systemic response to the primary tumor. We also identified elevated neuron-, oligodendrocyte-, and astrocyte-derived cfDNA in a subpopulation of patients with brain metastases compared with cancer patients without brain metastasis. Cell type–specific cfDNA methylation markers enable the identification of collateral tissue damage in cancer, revealing the presence of metastases in specific locations and potentially assisting in early cancer detection.

## Graphical abstract

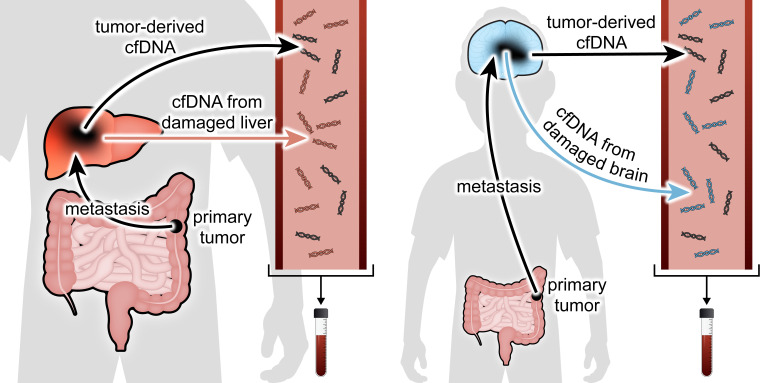

## Introduction

Cancer-induced damage to host tissues, both at the primary site and in metastatic locations, is a direct cause of cancer morbidity and mortality. Proposed mechanisms mediating this damage include inflammation (1), alteration of the microenvironment by tumor-secreted factors (2, 3), physical interactions between cancer cells and the microenvironment, and mechanical forces (4). The number of cells surrounding the tumor that undergo death by apoptosis or necrosis is unknown, and it is difficult to assess such damage to normal tissues surrounding cancer other than via histologic analysis. A minimally invasive method for sensitive detection of collateral tissue damage in cancer could reveal the presence of metastases, an essential component of cancer clinical evaluation. Such a method could also assist in early detection of primary cancer by sensing microenvironmental effects, on top of signals released by cancer cells.

We and others have recently described an approach for identifying the tissue sources of cell-free DNA (cfDNA), based on tissue-specific, stable, and universal methylation patterns in cfDNA. Such cell type–specific markers allow the inference of cell death in multiple settings, for example cardiomyocyte cell death following myocardial infarction and exocrine pancreas damage in pancreatic cancer and in pancreatitis (5–9). Notably, this approach does not rely on genetic differences between the host and the tissue of interest (as commonly used for detection of circulating tumor DNA; ref. 10) and hence can detect damage to genetically normal cells (11).

We hypothesized that cancer-triggered tissue damage releases cfDNA from injured cells of the host tissue and that such cfDNA could be identified and provide information on the existence and location of cancer. We focused on the setting of locally advanced disease and metastases, where the affected organ is genetically normal but epigenetically distinct from the tumor. We examined plasma taken from patients with liver or brain metastasis, patients with tumors that metastasized to other organs, patients with localized tumors, and healthy controls. We found that metastatic patients had elevated levels of cfDNA bearing the methylation markers of the metastasis host tissue — liver (hepatocytes) or brain (neurons, oligodendrocytes, astrocytes). In addition, patients with local pancreatic or colon cancer had elevated hepatocyte cfDNA, potentially reflecting liver damage due to bile duct obstruction (in the case of pancreatic cancer), the presence of micrometastases, or alternatively tumor-induced systemic toxic factors. We propose that this biologic phenomenon can be used for early detection of the presence and location of metastases, for monitoring progression of metastatic disease, and potentially for improving detection of early-stage cancer.

## Results

### Identification of hepatocyte-derived cfDNA in patients with cancer.

We recently described hepatocyte-specific DNA methylation markers (6). Using these markers, we determined plasma concentrations of liver-derived cfDNA in 268 individuals who were recruited to the study and signed an informed consent document: 65 healthy donors, 85 patients with localized cancer (outside the liver), 55 patients with metastatic cancer not involving the liver, and 63 patients with cancer with liver metastasis.

**Figure 1:**
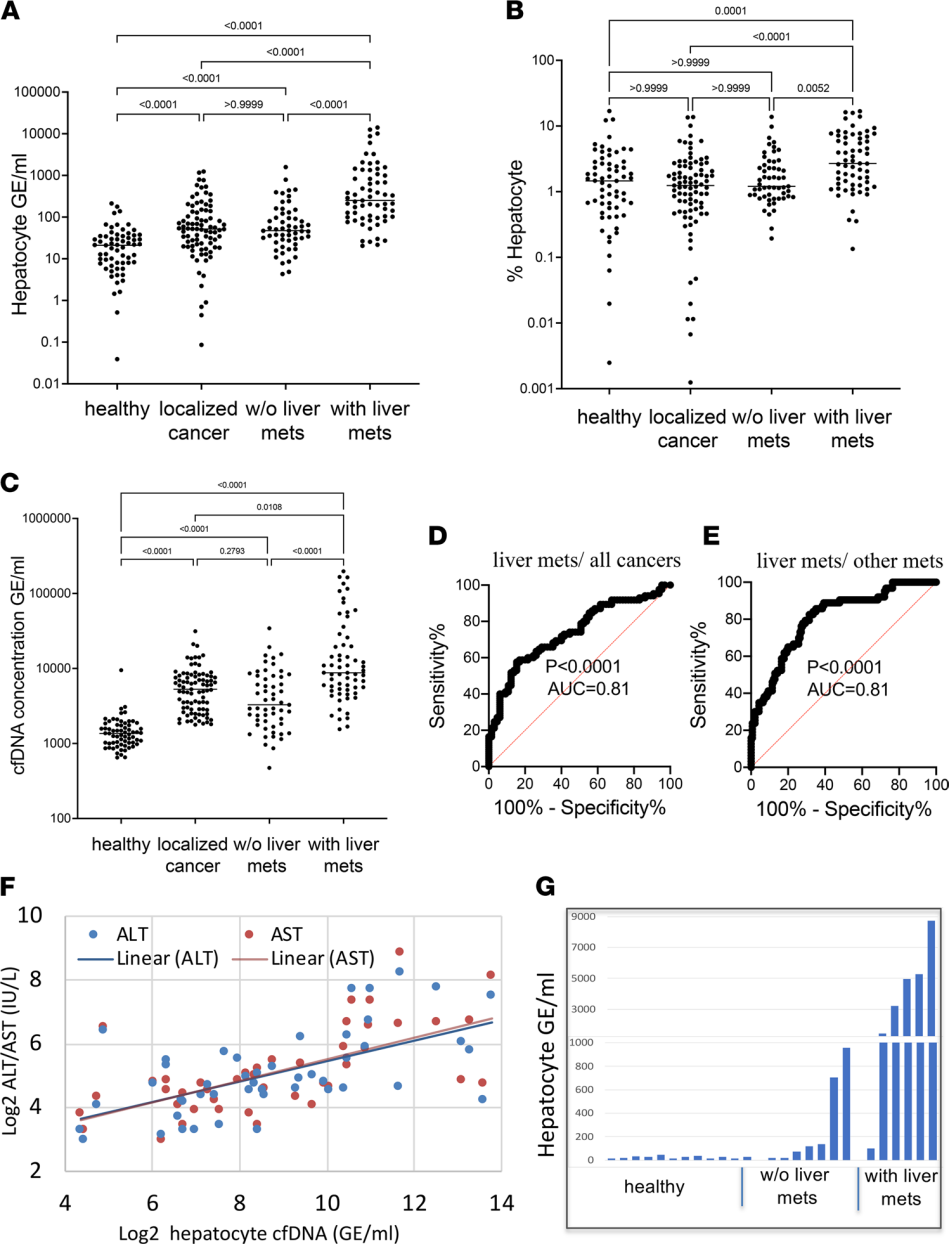
Hepatocyte cfDNA in patients with liver metastasis. (**A**) Hepatocyte cfDNA (average signal of 3 hepatocyte markers) in healthy controls (*n* = 65), localized cancer patients (*n* = 85), metastatic cancer patients with no liver metastasis (*n* = 55), and cancer patients with liver metastasis (*n* = 63). Each dot represents 1 plasma sample processed to extract cfDNA, treated with bisulfite, and PCR amplified and sequenced. The fraction of hepatocyte-derived cfDNA was multiplied by the total concentration of cfDNA per sample. Kruskal-Wallis test with Dunn’s post hoc correction for multiple comparisons. (**B**) Percentage of hepatocyte-derived cfDNA in the same plasma samples as in **A**. Kruskal-Wallis test with Dunn’s post hoc correction for multiple comparisons. (**C**) Total cfDNA levels in the same patients as in **A**. Kruskal-Wallis test with Dunn’s post hoc correction for multiple comparisons. (**D**) ROC curve for distinguishing cancer patients with liver metastases from other cancer patients (having localized or metastatic disease without liver involvement), based on the 3 hepatocyte cfDNA markers. AUC 0.81 (95% CI = 0.74 to 0.87), *P* < 0.0001. (**E**) ROC curve for distinguishing stage 4 cancer patients with or without liver metastases, using hepatocyte markers. AUC 0.81 (95% CI = 0.73 to 0.89), *P* < 0.0001. (**F**) Correlation between hepatocyte cfDNA levels and alanine transaminase (ALT, blue) or aspartate transaminase (AST, red) in cancer patients with liver metastases. Spearman’s correlation, ALT *r* = 0.6, *P* < 0.0001; AST *r* = 0.68, *P* < 0.0001. (**G**) Assessment of hepatocyte-derived cfDNA using data from Illumina 450K arrays, on an independent group (*n* = 12 healthy controls, 7 patients with metastatic cancer not involving the liver, and 6 patients with liver metastasis). Plasma samples were subjected to whole-methylome analysis using 450K arrays, and analyzed using an atlas of cell type–specific methylomes (13) (Methods). Hepatocyte cfDNA levels in cancer patients with liver metastasis compared with metastatic cancer patients with no liver metastasis; Wilcoxon’s *P* < 0.014.

Patients with liver metastases had more hepatocyte-derived cfDNA (measured by either the fraction of cfDNA derived from hepatocytes or the hepatocyte genome equivalents per milliliter) than healthy donors, patients with local cancer, or patients with non-liver-metastatic disease (median concentration of liver-derived cfDNA in healthy controls, 21 genome equivalents/mL [GE/mL]; in localized non-liver cancer patients, 52 GE/mL; in metastatic cancer patients with no liver metastases, 47 GE/mL; in cancer patients with liver metastases, 251 GE/mL) (Figure 1, A and B). Thus, cancer metastases to the liver caused hepatocyte damage, which was detectable in cfDNA. We also noticed that patients with cancer not involving the liver had on average higher levels of hepatocyte cfDNA than healthy people (see partial explanation below). Hepatocyte cfDNA was not different among patients with non-liver early-stage disease (local) and patients with metastatic disease not involving the liver. Total levels of cfDNA were higher in cancer patients with liver metastases compared with patients without liver metastases (Figure 1C).

We then assessed assay performance in discriminating patients with cancer with liver metastasis from other cancer patients, an exercise mimicking a clinical attempt to determine if a patient with newly diagnosed cancer has liver metastases. We plotted receiver operating characteristic (ROC) curves and found that the area under the curve (AUC) was 0.81 (95% CI = 0.74 to 0.87, *P* < 0.0001) for hepatocyte cfDNA concentration, indicating high sensitivity and specificity (Figure 1D). Using a cutoff of 561 GE/mL of liver-derived cfDNA, the specificity and sensitivity for detecting liver metastases were 95% and 35%, respectively. Moreover, hepatocyte cfDNA levels were able to distinguish patients with stage 4 cancer with and without liver metastases (AUC = 0.81, 95% CI = 0.73 to 0.89, *P* < 0.0001) (Figure 1E).

We explored the correlation between the concentration of liver-derived cfDNA and markers of liver damage, circulating aspartate transaminase (AST) and alanine transaminase (ALT). As previously reported, AST and ALT levels did not correlate with hepatocyte cfDNA in healthy controls (6). However, a comparison of AST and hepatocyte cfDNA levels in 45 patients with cancer with liver metastases (for whom we had liver function tests) yielded Spearman’s correlation value of 0.68 and *P* < 0.0001. Similarly, a comparison of ALT to hepatocyte cfDNA levels in 47 patients with cancer with liver metastases yielded Spearman’s correlation coefficient of 0.6 and *P* < 0.0001, supporting the validity of hepatocyte cfDNA methylation markers in the setting of liver metastasis (Figure 1F). We further examined the ability of AST, ALT, or both markers combined to differentiate patients with liver metastases from patients with local disease and from patients with metastases not involving the liver. The AUC for these ROC curves was around 0.67, indicating that these markers are inferior to hepatocyte cfDNA for detection of liver metastases (Supplemental Figure 1; supplemental material available online with this article; https://doi.org/10.1172/jci.insight.153559DS1). Furthermore, a combined assay taking into account both liver cfDNA and liver enzymes had a lower performance than a liver cfDNA-based assay (AUC ~0.7), probably due to the lower specificity of AST/ALT as indicators of liver damage compared with hepatocyte cfDNA markers, as we have shown before (6) (Supplemental Figure 1).

To provide further support to the finding that hepatocyte cfDNA reflects liver damage in metastatic disease, we assessed the presence of liver-derived cfDNA in metastatic patients using an independent methodology and an independent cohort of patients, relying on a wider range of CpG sites represented in the Illumina 450K BeadChip array. We selected a subset of 115 hepatocyte-specific CpG sites (102 hypomethylated, 13 hypermethylated in hepatocytes) and used them to estimate the contribution of liver-derived cfDNA by deconvolution. We used plasma samples from an independent cohort of patients, whose samples were analyzed using Illumina 450K BeadChip arrays. The plasma deconvolution results revealed significantly higher levels of hepatocyte cfDNA in patients with cancer with liver metastasis compared with metastatic cancer patients with no liver metastasis (*n* = 12 healthy controls, 7 patients with metastatic cancer not involving the liver and 6 patients with liver metastasis; Wilcoxon’s *P* < 0.014, Figure 1G), further supporting the idea that metastases to the liver cause the release of detectable cfDNA from hepatocytes. As with the directed assay shown in Figure 1A, the methylome-wide analysis showed that patients with cancer without liver metastases had higher-than-normal levels of hepatocyte cfDNA (Figure 1G) (see discussion below).

### Evidence for liver damage in pancreatic and colon cancer.

**Figure 2:**
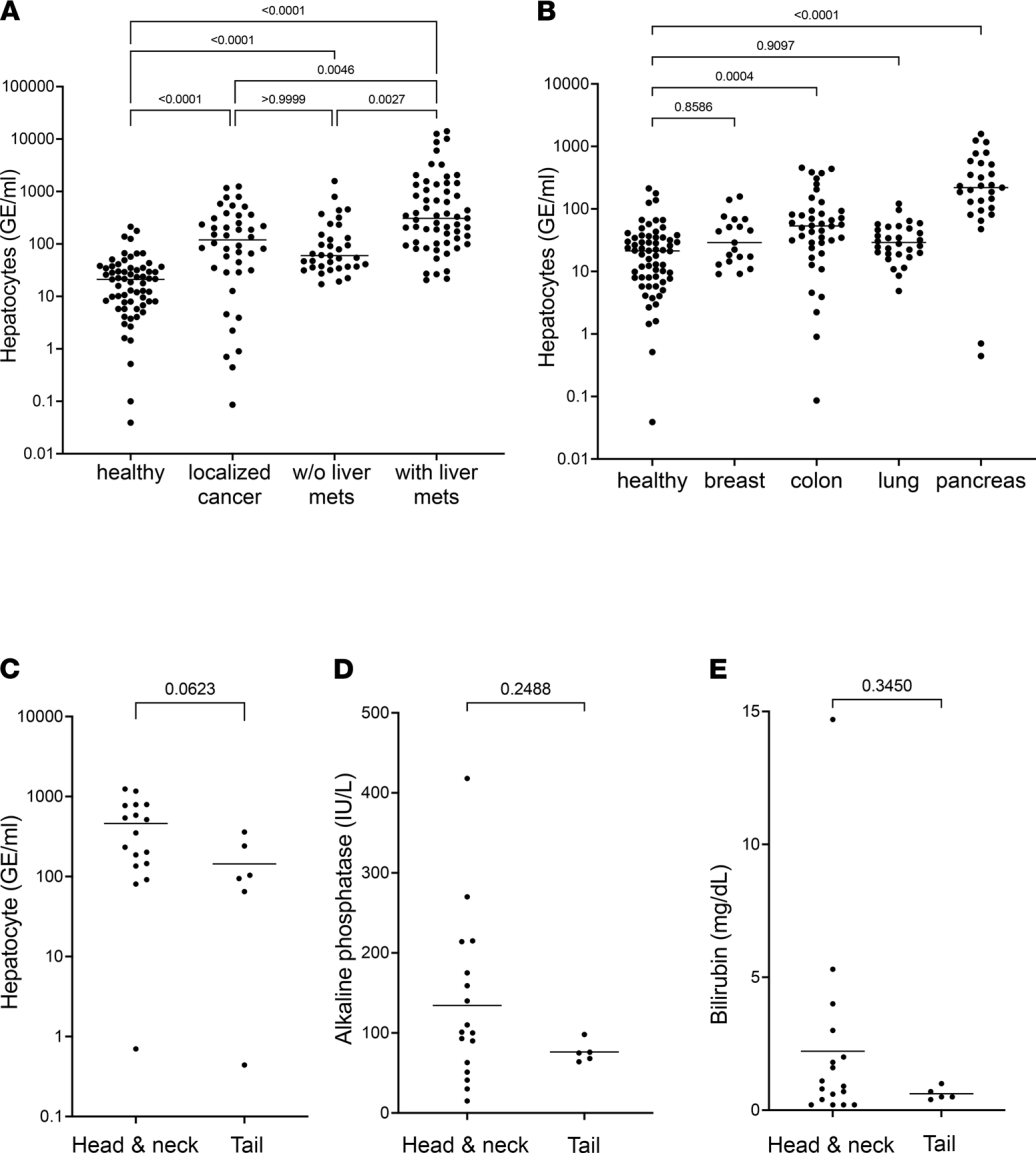
Hepatocyte cfDNA in treatment-naive patients and in patients with different primary and metastatic cancers. (**A**) Hepatocyte cfDNA in treatment-naive patients. Statistical significance was measured by Kruskal-Wallis test with Dunn’s post hoc correction for multiple comparisons. (**B**) Hepatocyte cfDNA in patients with cancer after breakdown by tissue of origin. Healthy controls (*n* = 65), breast (*n* = 19), colon (*n* = 42), lung (*n* = 33), pancreas (*n* = 33). Statistical significance was measured by Kruskal-Wallis test with Dunn’s post hoc correction for multiple comparisons. (**C**) Hepatocyte cfDNA in patients with pancreatic cancer after breakdown by tumor anatomic location. Pancreas head (*n* = 16) or neck (*n* = 1), versus pancreas tail (*n* = 6), Mann-Whitney *U* test. (**D** and **E**) Alkaline phosphatase (**D**) and bilirubin (**E**) in pancreatic cancer patients after breakdown to tumor anatomic location. Pancreas head (*n* = 16) or neck (*n* = 1), versus pancreas tail (*n* = 5), Mann-Whitney *U* test.

In attempting to explain the abnormally high levels of liver cfDNA in cancer patients who did not have liver metastases, we excluded patients who had been treated prior to blood sampling to rule out drug toxicity (65 healthy donors; 42 patients with localized cancer, excluding liver cancer; 35 patients with metastatic cancer not involving the liver; and 59 cancer patients with liver metastasis). Hepatocyte cfDNA was also elevated in treatment-naive cancer patients with no involvement of the liver, demonstrating that elevated hepatocyte cfDNA in cancer is a biologic, not an iatrogenic, phenomenon (Figure 2A).

We then examined our samples, taking into account the location of the primary cancer. Our cohort consisted mostly of tumors of the colon, lung, pancreas, and breast. Strikingly, samples from patients with pancreatic cancer had the highest concentrations of hepatocyte cfDNA. Samples from patients with colon cancer also had higher-than-normal hepatocyte cfDNA (Figure 2B). We further studied the patients with pancreatic cancer, attempting to understand the underlying process accounting for the evidence of liver damage. Elevated hepatocyte cfDNA was seen in patients with either local or metastatic pancreatic cancer (Supplemental Table 1). We hypothesized that elevated hepatocyte cfDNA in patients with pancreatic cancer could result from the presence of liver micrometastases among patients thought to have local disease, from a local effect whereby the growing tumor obstructs the bile duct and indirectly leads to liver damage, or alternatively from a systemic effect, e.g., toxic factors released by the primary tumor. Discriminating between these possibilities is challenging. If hepatocyte cfDNA is due to local damage, patients with tumors localized in the head or neck of the pancreas should show more hepatocyte cfDNA than patients with tail tumors. However, head tumors are also more likely to metastasize to the liver than tail tumors (12). Thus, both a local damage model and a micrometastasis model predicted higher levels of liver cfDNA in patients with tumors in the head of the pancreas. Indeed, patients with local head or neck tumors trended to higher levels of hepatocyte cfDNA than patients with local tail tumors (*P* = 0.06, Figure 2C). To further test the hypothesis that liver cfDNA in pancreatic head tumors reflects bile duct obstruction, we determined the levels of circulating bilirubin and alkaline phosphatase as markers for bile duct damage. There were no differences in bilirubin or alkaline phosphatase levels in patients with head/neck tumors compared to tail tumors, arguing against prominent bile duct damage (Figure 2, D and E). We were able to obtain follow-up records for 26 patients with pancreatic cancer who were considered to have localized disease at the time of sampling. Of these, 5 patients developed later liver metastases. They did not have, as a group, higher levels of hepatocyte-derived cfDNA (not shown). Additional studies are needed to determine the root cause of elevated hepatocyte cfDNA in some patients with pancreatic or colon cancer not known to have liver metastases.

### Identification of brain-derived cfDNA in patients with cancer with brain metastases.

**Figure 3:**
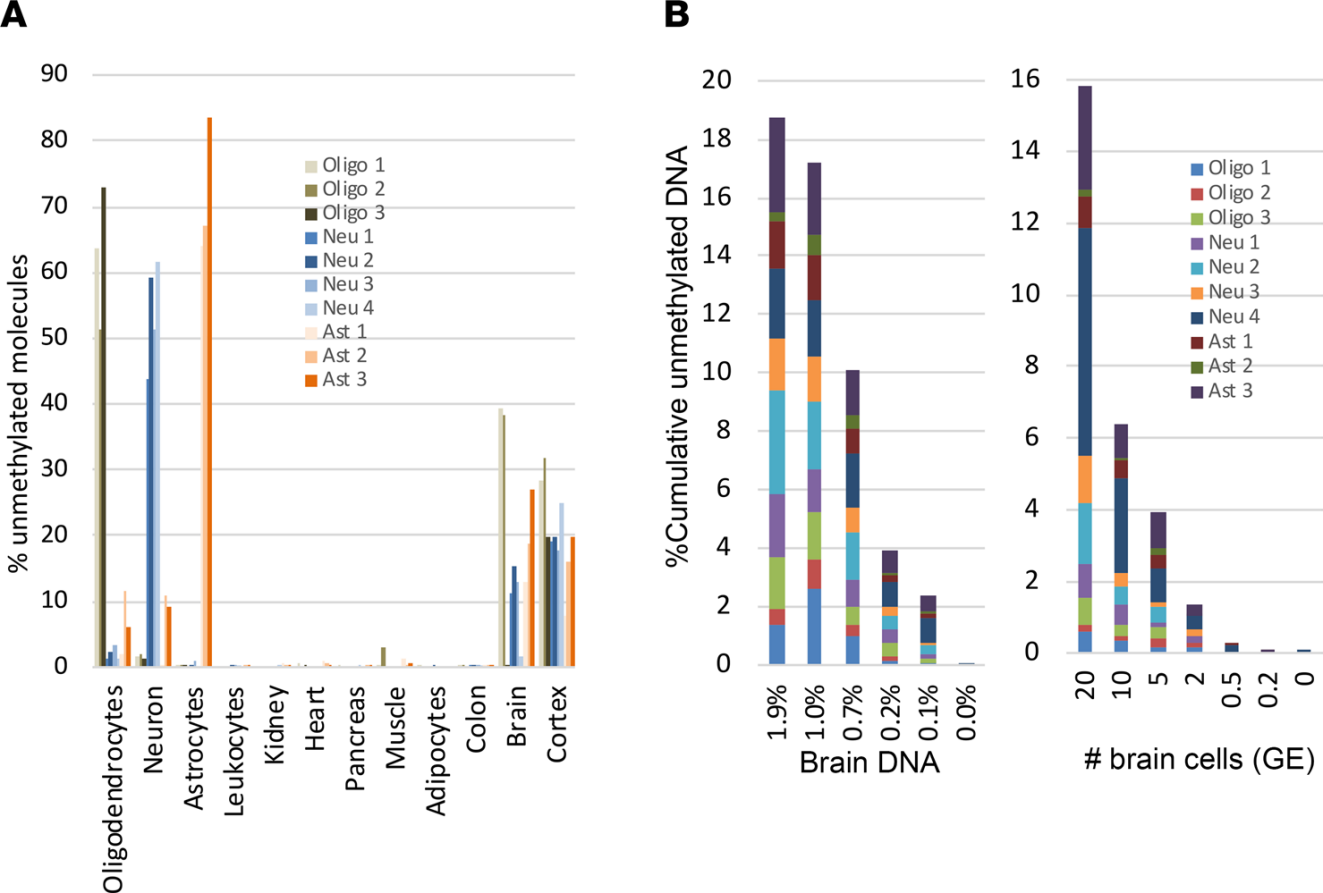
Specificity and sensitivity of brain methylation markers. (**A**) Methylation status of 10 brain-derived markers in genomic DNA from multiple human tissues. Each color represents a different locus that is differentially hypomethylated in a specific brain cell type. Shown is the methylation score of multiple CpG sites in each block (i.e., the fraction of molecules that are fully unmethylated in a given sample). (**B**) Sensitivity of brain-derived methylation markers. Brain DNA was spiked into leukocyte DNA as indicated, and the fraction of brain DNA was assessed using bisulfite conversion, multiplex PCR amplification of brain markers, and sequencing. Left, 20 brain cell GE (120 pg brain DNA) were mixed with blood DNA (0 to 10 ng). Right, brain cell DNA (20 to 0.2 GE) was diluted into 10 ng of blood DNA.

We recently described the identification of tissue-specific methylation markers through a comparison of extensive genome-wide DNA methylation data sets based on Illumina Infinium Human Methylation 450K BeadChip arrays (13, 14). We used publicly available methylation profiles from The Cancer Genome Atlas and Gene Expression Omnibus repositories, along with data that we generated locally. Using this comparative analysis, we selected 10 genomic loci, which are unmethylated specifically in neurons (4 markers), oligodendrocytes (3 markers), or astrocytes (3 markers) and methylated in all other examined cell types. Genomic sequences of primers are detailed in Supplemental Table 1. To test our in silico predictions regarding marker specificity, we applied bisulfite treatment, multiplex PCR, and next-generation sequencing to determine the methylation status of each marker in a panel of DNA samples obtained from multiple human tissues, as previously described (15) (Figure 3A). To determine assay sensitivity, we serially diluted brain DNA into leukocyte DNA and found that the markers allowed the detection of as little as 0.1% brain DNA in a mixture, or just 1 brain genome in a mixture of 1000 genomes (Figure 3B).

**Figure 4:**
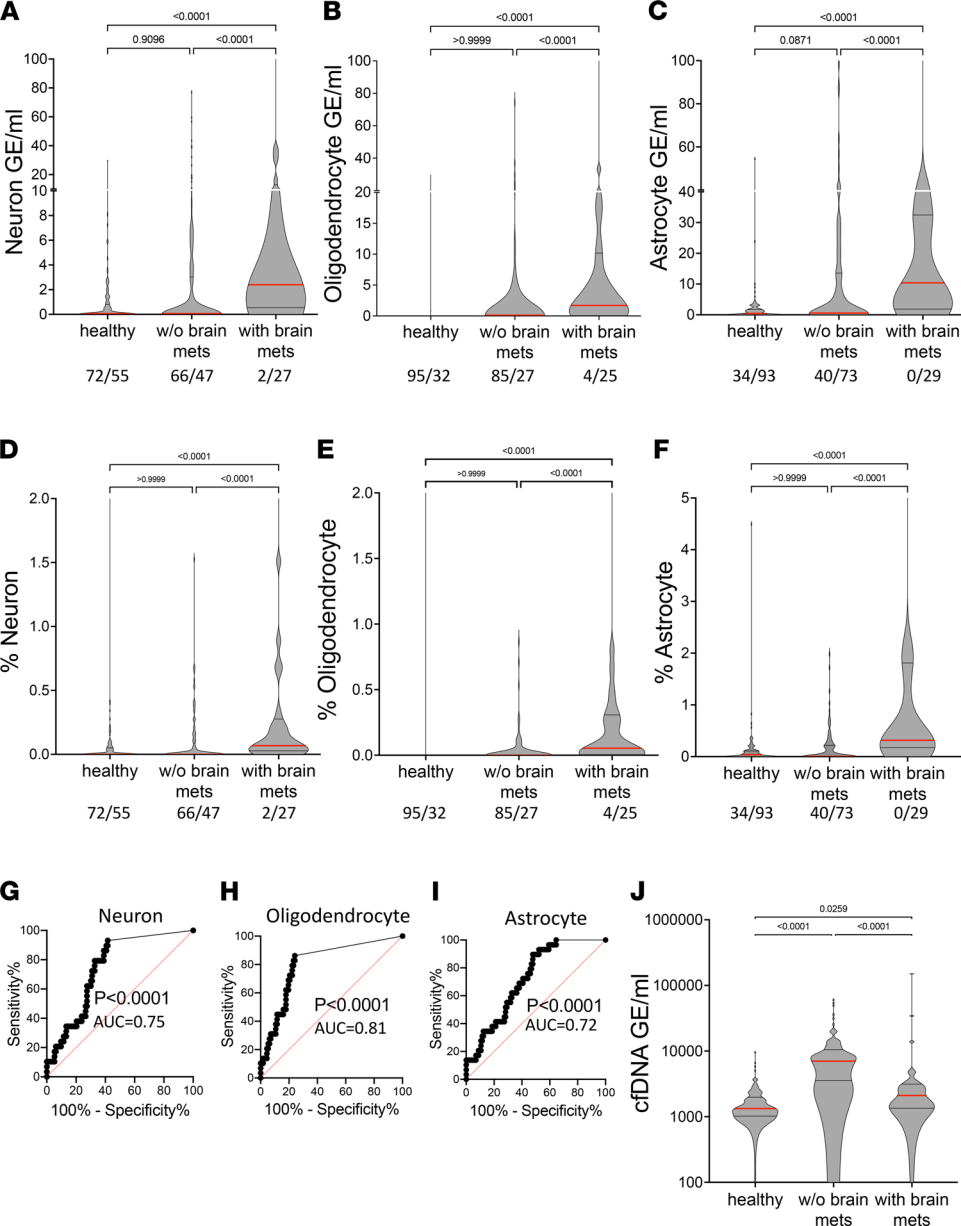
Plasma concentrations of brain-derived cfDNA. (**A**–**C**) Brain cfDNA levels in healthy controls (*n* = 127), cancer patients (localized and non-brain-metastatic, *n* = 113), and cancer patients with metastases to the brain (*n* = 29). Shown are the average levels in plasma of 4 neuronal markers (**A**), 3 oligodendrocyte markers (**B**), and 3 astrocyte markers (**C**). Each dot represents 1 plasma sample. Numbers in the figure indicate samples with 0/above 0 cfDNA molecules with a brain-derived signature. Statistical significance was measured by Kruskal-Wallis test with Dunn’s post hoc correction for multiple comparisons. (**D**–**F**) Brain cfDNA levels as in **A**–**C**, expressed as percentage of cfDNA derived from the indicated brain cell type. Statistical significance was measured by Kruskal-Wallis test with Dunn’s post hoc correction for multiple comparisons. (**G**–**I**) ROC curve for the diagnosis of brain collateral damage in plasma of cancer patients with brain metastasis compared to cancer patients without brain metastases. (**G**) Neuronal markers; AUC 0.75, 95% CI = 0.66 to 0.84; *P* < 0.0001. (**H**) Oligodendrocyte markers; AUC 0.81; 95% CI = 0.72 to 0.89; *P* < 0.0001. (**I**) Astrocyte markers; AUC 0.72, 95% CI = 0.63 to 0.81; *P* < 0.0001. (**J**) Plasma concentrations of total cfDNA in the same donors as in **A**–**C**. Statistical significance was measured by Kruskal-Wallis test with Dunn’s post hoc correction for multiple comparisons.

We then determined the plasma concentrations of brain-derived cfDNA (including methylation markers of neurons, astrocytes, and oligodendrocytes) in 269 samples: 127 from healthy controls, 113 from cancer patients with no brain metastasis, and 29 from cancer patients with brain metastasis. Strikingly, cfDNA levels from each of the 3 brain cell types were significantly higher in cancer patients with brain metastases compared with healthy controls or with cancer patients (localized and metastatic combined) without brain metastases (*P* < 0.001). Elevated brain-derived cfDNA was seen when measuring either its absolute concentration (Figure 4, A–C) or its fraction (Figure 4, D–F).

Most patients with brain metastases had small but measurable levels of brain-derived cfDNA, as opposed to healthy controls and patients with cancer without brain metastases, who typically had no signal at all (Figure 4, A–F). A small subpopulation of patients with brain metastases had extremely high levels of brain-derived cfDNA. Clinical characterization of these patients did not explain their unique cfDNA phenotype and the heterogeneity observed.

To determine how well brain-derived cfDNA markers can distinguish patients with brain metastases from cancer patients without brain involvement, we generated ROC curves for the signal from each of the brain cell types. The markers of each brain cell type were able to identify the plasma of patients with brain metastases with an AUC of 0.72–0.81 (Figure 4, G–I). The sensitivity at 95% specificity was 17.2% for neuron markers, 13.8% for astrocyte markers, and 20.7% for oligodendrocyte markers.

Unlike brain-derived cfDNA, the total concentration of cfDNA circulating in plasma of cancer patients did not correlate with presence or absence of brain metastases (Figure 4J). On the contrary, plasma samples from patients with brain metastases had significantly lower levels of total cfDNA compared with other cancer patients (*P* < 0.0001). Given that the relative contribution of tumor-derived DNA to the total cfDNA pool is typically very low, this phenomenon is likely related to altered turnover of immune cells, which contribute the majority of cfDNA. It is also possible that patients with brain metastases tend to die earlier, with an overall tumor load (and associated effects on immune-derived cfDNA) that is lower compared with other patients with cancer.

These findings indicate that brain damage caused by metastatic seeding from other tissues may result in the release of detectable cfDNA from multiple brain cell types. Tumor heterogeneity with regard to this phenomenon merits further investigation.

### Collateral damage signal does not result from aberrant tumor methylation.

Aberrant DNA methylation has been recognized in many pathologies, including cancer (16, 17). Cancer-associated changes in DNA methylation include global hypomethylation and hypermethylation (18), and thus dying cancer cells could in theory contribute hypomethylated cfDNA molecules to the plasma, which might be misdiagnosed as emerging from specific tissues such as the brain or liver. We therefore determined the status of our brain and liver methylation markers in multiple cancer methylomes available at The Cancer Genome Atlas (TCGA). All CpG sites that constituted part of our brain cell type markers (neuron/oligodendrocyte/astrocyte markers) and were reported by TCGA (covered by the Illumina 450K BeadChip array) were unmethylated in DNA from brain or a specific brain cell type, respectively, and highly methylated in multiple normal tissues, as expected. Importantly, all marker CpGs were methylated to the same extent in multiple tumors (14 normal tissue types, 17 tumor types, total 54 samples). Similarly, all 5 CpG sites that constitute part of our hepatocyte markers and were reported by TCGA (covered by the Illumina 450K BeadChip array) were unmethylated in DNA from liver and hepatocytes, highly methylated in multiple normal tissues, and methylated to the same extent in multiple tumors (12 normal tissue types, 18 tumor types, total 53 samples) (Supplemental Figure 2).

We further investigated all the CpGs included in amplicons of our markers utilizing data from whole-genome bisulfite sequencing. This approach allowed us to cover all the CpG sites that constitute our brain and hepatocyte markers. The same pattern appeared: all marker CpG sites were unmethylated in DNA from brain or liver, highly methylated in all other normal tissues, and methylated to the same extent in multiple tumors (Supplemental Figure 3).

These analyses strongly suggest that liver and brain methylation markers observed in cfDNA from cancer patients indeed derive from the liver and brain, rather than from aberrant methylation of the tumor itself.

## Discussion

Our results show that collateral tissue damage caused by metastatic tumors causes the release of cfDNA from affected tissues to circulation, which can be detected using tissue-specific methylation markers. We demonstrate the presence of hepatocyte- and brain-derived cfDNA in patients with cancer with liver and brain metastases, respectively.

Several lines of evidence support the specificity of the cfDNA signal for brain or liver tissue: (i) methylation patterns of our markers in multiple human tissues; (ii) a correlation to clinical data, showing that some patients with brain metastasis have more brain cfDNA than cancer patients with no brain involvement and that hepatocyte cfDNA levels are higher in patients with liver metastasis than in patients with metastases to other organs; (iii) a correlation between hepatocyte cfDNA in the metastatic liver setting and the plasma levels of liver enzymes AST and ALT; (iv) the identification of selective elevation of liver cfDNA in patients with liver metastases using both a directed approach (selected markers amplified by PCR) and a plasma methylome analysis involving multiple informative CpGs; and (v) the demonstration that the methylation markers used do not change their pattern of methylation in tumors, i.e., they retain their tissue specificity.

We note that our current findings do not reveal why host tissues release elevated levels of cfDNA. In fact, elevated brain or liver cfDNA could in principle reflect impairment of phagocytosis in these organs, or a disruption of the blood-brain barrier in the case of elevated brain cfDNA, rather than elevated cell death; nonetheless, elevation of brain- and liver-specific cfDNA is still an indication of abnormality in the host tissue inflicted by cancer metastases. Recently described technologies for noninvasive inference of gene expression patterns from cfDNA may help address the biology of host tissue prior to cell death (19).

We also note a significant elevation of hepatocyte cfDNA in patients with pancreatic and colon cancer without known liver metastases. Some potential reasons for liver damage in patients diagnosed as having local cancer are mechanical obstruction of the bile duct by the growing tumor, often reflected in jaundice and elevated liver enzymes AST and ALT (relevant only to pancreatic cancer) (20); secreted factors derived from the tumor bed reaching the liver through the portal circulation (21), potentially related to the formation of a prometastatic niche (22); the presence of liver micrometastases in cancer patients that is considered local based on imaging; or cancer-related impairment of clearance of naturally dying liver cells, leading to elevated release of DNA to the circulation. We ruled out tissue damage due to chemotherapy or irradiation, since elevated hepatocyte cfDNA was also seen in patients who were sampled prior to treatment. It does remain possible, though, that hepatocyte cfDNA was elevated because of use of over-the-counter medications such as pain relivers (e.g., paracetamol). Discriminating between these possibilities is challenging and will likely require long-term follow-up of patients to determine if the presence of liver cfDNA in early-stage cancer is predictive of future liver metastases, or whether it correlates with other clinical or biochemical features.

The presence of cfDNA markers of metastasis could find utility in liquid biopsies. A blood test for the presence of metastatic disease, potentially even suggesting the tissue location of metastases, can help clinical evaluation and decisions regarding treatment. In addition, the idea that cancer-induced collateral damage is reflected in cfDNA methylation patterns also applies to the detection of primary cancer. A growing carcinoma may cause death (and hence release of detectable cfDNA signal) in normal adjacent epithelial or even stromal cells. The signals from damaged “normal adjacent” cells could be detected using cell type–specific methylation markers (but not mutation markers) and increase the sensitivity of methylation-based analysis for early detection of cancer.

## Methods

### Clinical samples.

Samples were obtained from patients treated in these centers: The Hebrew University-Hadassah Medical Center, Jerusalem, Israel; Pulmonary Institute, Shaare Zedek Medical Center, Jerusalem, Israel; and the Department of Medical Oncology, Dana-Farber Cancer Institute and Harvard Medical School, Boston, Massachusetts, USA. In addition, samples, genomic data, and health information were obtained from the Partners HealthCare Biobank, a biorepository of consented patients’ samples at Partners HealthCare (parent organization of Massachusetts General Hospital and Brigham and Women’s Hospital). For the directed approach (selected markers amplified by PCR), a total of 192 adults recruited from the same centers participated in the study as unpaid healthy controls. The age distribution of these healthy donors was 18–85 years, average 40.9. The sex distribution was 120 women, 71 men; for 1 donor this information was not available. All donors serving as controls denied having any acute or chronic illnesses or receiving any medications at the time of blood donation. For the plasma methylome analysis involving multiple informative CpGs, 25 adults participated in the study, including 12 healthy controls recruited from the same centers as the patients. Patient demographics, clinical data, and cfDNA data are detailed in Supplemental Table 1.

### cfDNA PCR-sequencing analysis.

Blood samples were collected in EDTA tubes and centrifuged at 1500*g* for 10 minutes at 4°C within 2 hours of collection. Plasma was removed and recentrifuged at 3000*g* for 10 minutes at 4°C to remove any remaining cells. Plasma was then stored at −80°C until assay. cfDNA was extracted using the QIAsymphony SP instrument and its dedicated QIAsymphony Circulating DNA Kit (QIAGEN) according to the manufacturer’s instructions. DNA concentration was measured using the Qubit dsDNA HS Assay Kit (Thermo Fisher Scientific). cfDNA was treated with bisulfite using EZ DNA Methylation-Gold (Zymo Research) and PCR amplified with primers specific for bisulfite-treated DNA but independent of methylation status at the monitored CpG sites. Treatment with bisulfite led to degradation of 60%–90% of the DNA (on average, 75% degradation), consistent with previous reports (23). Note that while DNA degradation does reduce assay sensitivity (since fewer DNA molecules are available for PCR amplification), it does not significantly harm assay specificity since methylated and unmethylated molecules are equally affected. Primers were bar-coded using TruSeq Index Adapters (Illumina), allowing the mixing of samples from different individuals when sequencing PCR products using NextSeq sequencers (Illumina). Sequenced reads were separated by barcode, aligned to the target sequence, and analyzed using custom scripts written and implemented in R. Reads were quality filtered based on Illumina quality scores and identified by having at least 80% similarity to target sequences and containing all the expected CpGs in the sequence. CpGs were considered methylated if CG was read and were considered unmethylated if TG was read. The fraction of unmethylated molecules in a sample was multiplied by the total concentration of cfDNA in the sample, to assess the number of brain/liver genome equivalents per milliliter of plasma. The concentration of cfDNA was measured prior to bisulfite conversion, rendering the assay robust to potential intersample fluctuations in the extent of bisulfite-induced DNA degradation.

### Array-based analysis of plasma samples.

Hepatocyte-specific CpG sites were selected by examining whole-genome bisulfite sequencing data and identifying differentially methylated or differentially unmethylated regions. For hypermethylated markers, we selected regions showing a difference greater than 0.5 between the 75th percentile among the hepatocyte samples and the 5th percentile of all nonhepatocyte samples. For hypomethylated markers, a difference of 0.5 between 95th and 20th percentiles was required. The fraction of hepatocyte-derived cfDNA in a plasma sample, denoted by *p*, was estimated as the average estimation across all sites. For the hypermethylated sites, the estimation is the β value, and for hypomethylated sites it is 1 – β. The total hepatocyte-derived GE contribution to the cfDNA is *p* × (ng/mL) × 330.

### Code and data availability.

Custom script for sequence analysis is available from the authors upon reasonable request. All relevant data, including primer sequences, detailed PCR conditions, and additional protocols, are available from the authors upon reasonable request.

### Statistics.

To assess the significance of differences between groups, we used a 2-tailed Mann-Whitney *U* test. For multiple-group associations, the Kruskal-Wallis test with Dunn’s post hoc correction for multiple comparisons was performed. We calculated all CIs at the 95% level, and *P* value was considered significant when less than 0.05. Data are shown as medians in Figure 1, A and C, and Figure 2A (hepatocyte cfDNA with significant outliers) and in all other figures as means.

### Study approval.

This study was conducted according to protocols approved by the institutional review boards at each study site: Hadassah-Hebrew University Medical Center, Jerusalem, Israel; Shaare Zedek Medical Center, Jerusalem, Israel; Dana-Farber Cancer Institute and Harvard Medical School, Boston, Massachusetts, USA. In addition, samples, genomic data, and health information were obtained from the Partners HealthCare Biobank, a biorepository of consented patients’ samples at Partners HealthCare (parent organization of Massachusetts General Hospital and Brigham and Women’s Hospital).

Procedures were performed in accordance with the Declaration of Helsinki. Blood was obtained from donors who provided written informed consent. Case report forms were filled out by donors detailing underlying diseases.

## Author contributions

The study was conceptualized by YD, RS, and AL with input from BG. Experiments were performed by AL, SP, BO, and J Magenheim. Patient samples were collected by AZ, MM, AG, AK, CM, JEC, TP, AH, MT, AS, MFL, LB, KN, AR, SAP, MG, and SGG. Support for experimental design was provided by HZ, DN, RLW, and KLS. Bioinformatic analysis was performed by AL, J Moss, DC, NL, and TK. Analysis of data was done by AL, DN, HZ, and RS. Figures were prepared by AL and DN. The manuscript was written by AL, RS, and YD. The manuscript was reviewed with significant revisions by BG, RM, BMW, and AZ. The authors read and approved the final manuscript.

## Supplementary Material

Supplemental data

Supplemental table 1

## Version 1 01/25/2022

Electronic publication

## Version 2 01/26/2022

A spelling error was corrected in the abstract

## Version 3 01/27/2022

A correction was made to the author affiliation section
