# Supplementary material for: Liquid biopsy reveals collateral tissue damage in cancer
Source: JCI Insight. 2022 Jan 25;7(2):e153559. doi: 10.1172/jci.insight.153559 (PMC8855834; doi:10.1172/jci.insight.153559)
Supplement: Supplemental data [file jciinsight-7-153559-s212.pdf]

Supplemental Figure S1 ***: Utility of liver enzymes for distinguishing cancers with liver metastases from local cancers (left) and from metastatic cancer without involvement of the liver (right), using ROC curves.*** *A. ALT. B. AST. C. Combined ALT/AST. D. Combined ALT/AST and hepatocyte cfDNA.*

A

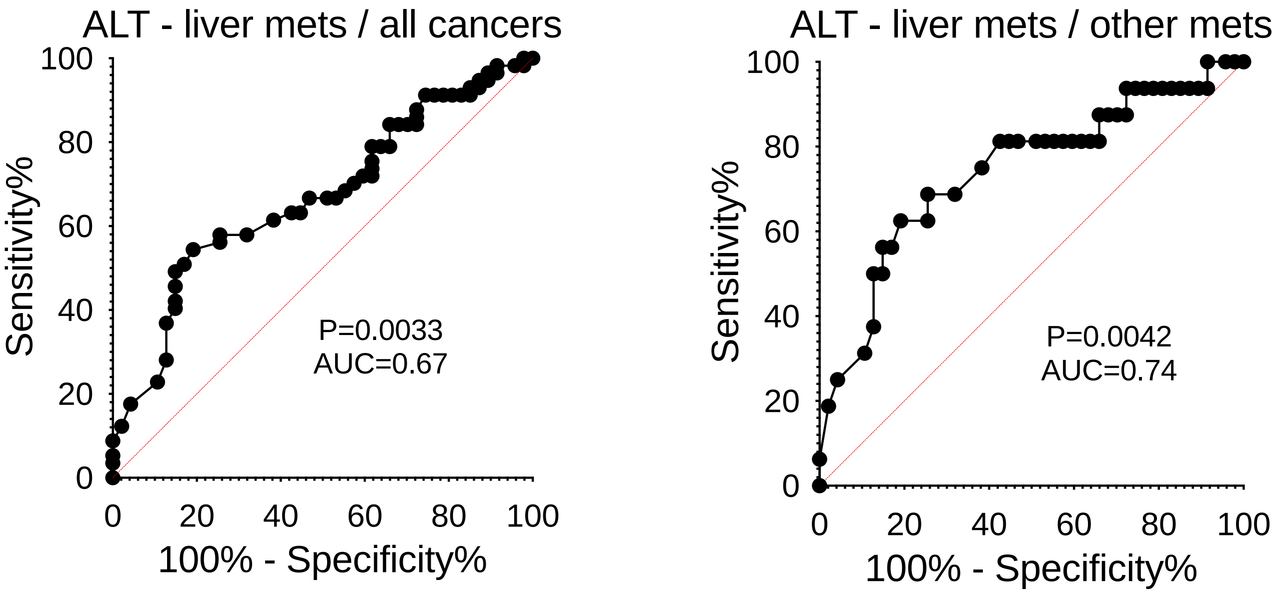

B

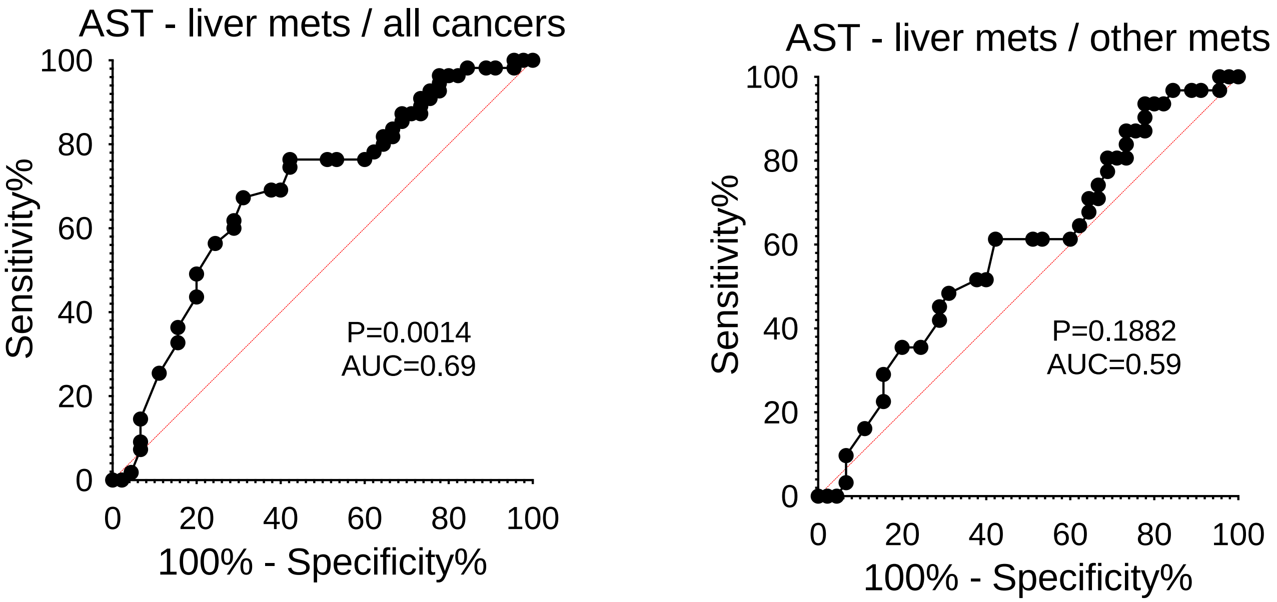

C

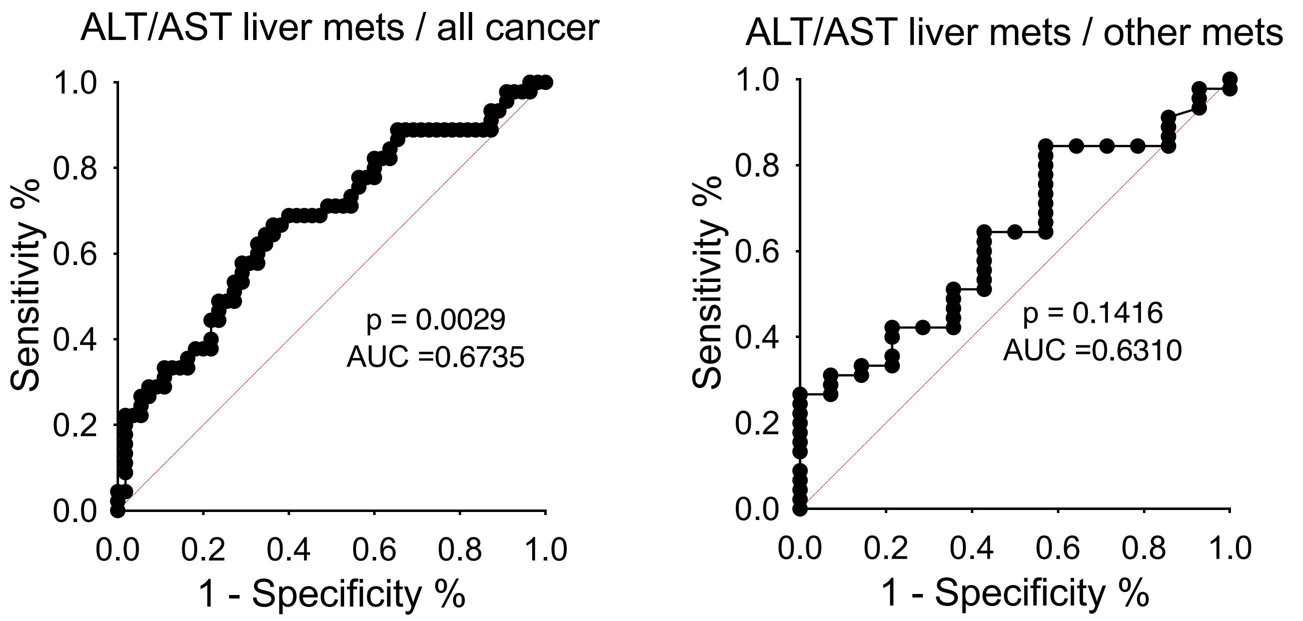
ALT/AST liver mets / all cancer
Sensitivity %
1 - Specificity %
p = 0.0029
AUC =0.6735
0.0
0.2
0.4
0.6
0.8
1.0
ALT/AST liver mets / other mets
Sensitivity %
1 - Specificity %
p = 0.1416
AUC =0.6310

D

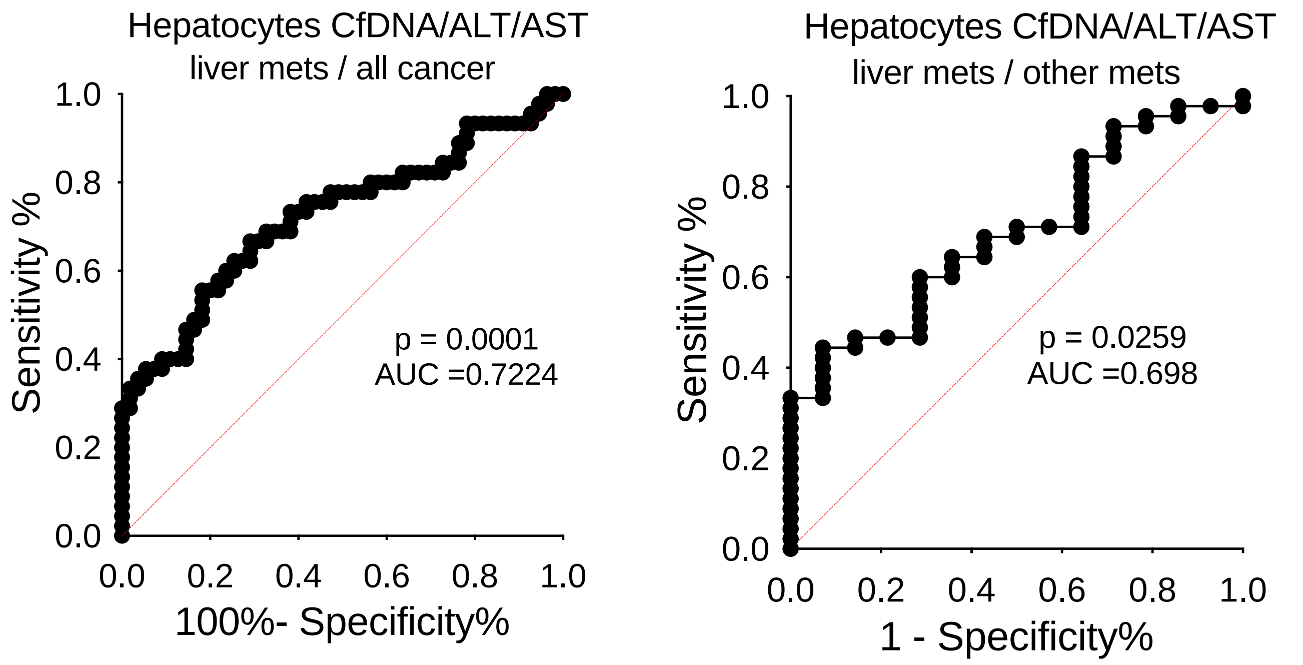
Hepatocytes CfDNA/ALT/AST
liver mets / all cancer
Sensitivity %
100%- Specificity%
p = 0.0001
AUC =0.7224
0.0
0.2
0.4
0.6
0.8
1.0
Hepatocytes CfDNA/ALT/AST
liver mets / other mets
Sensitivity %
1 - Specificity%
p = 0.0259
AUC =0.698

Supplemental Figure S2 : *Methylation of tissue-specific markers described in this study, using CpG sites covered by the Illumina Infinium 450k BeadChip array*. *A. Neuron markers. B. Oligodendrocyte markers C. Astrocyte markers D. Hepatocyte markers*

A

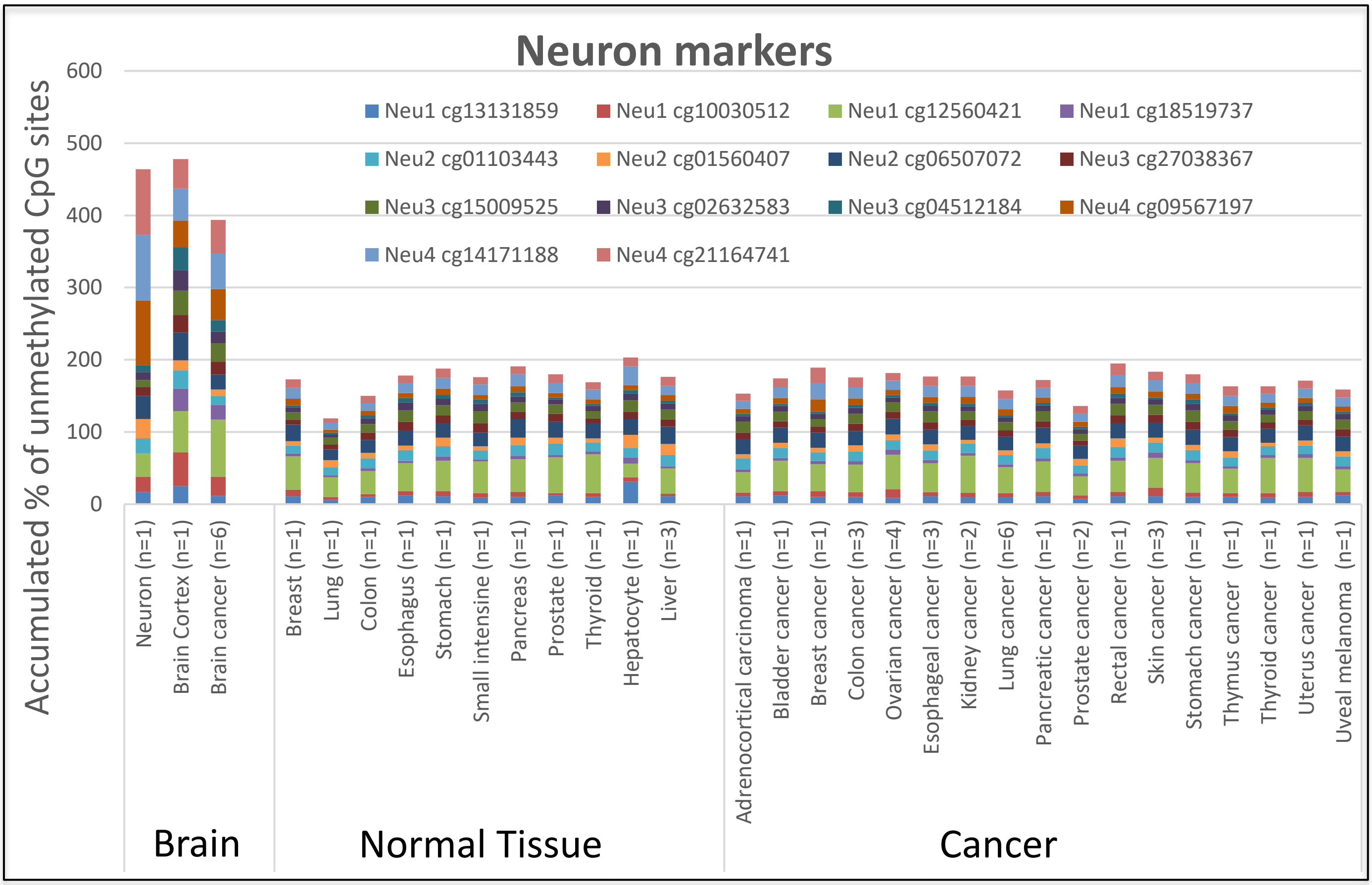

B

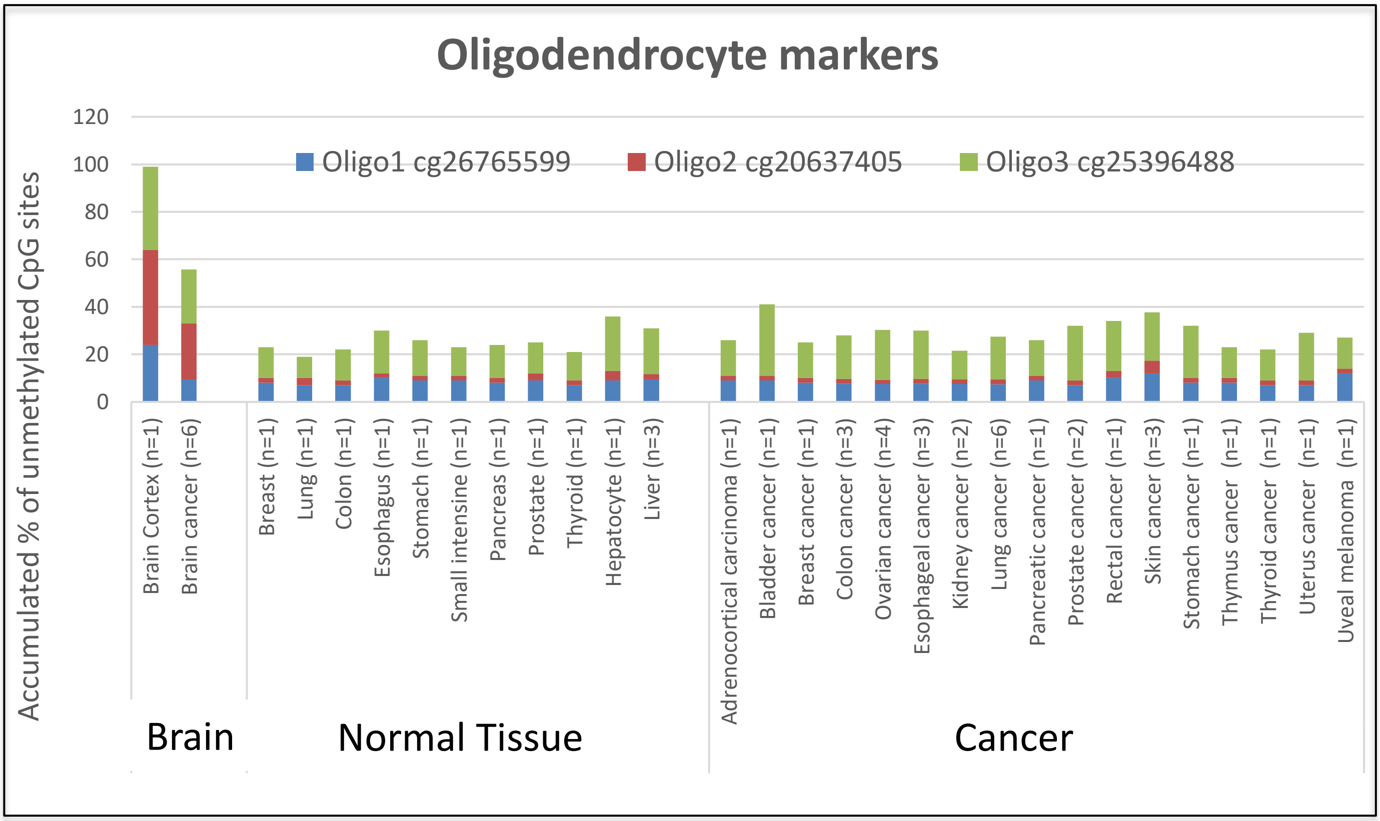

C

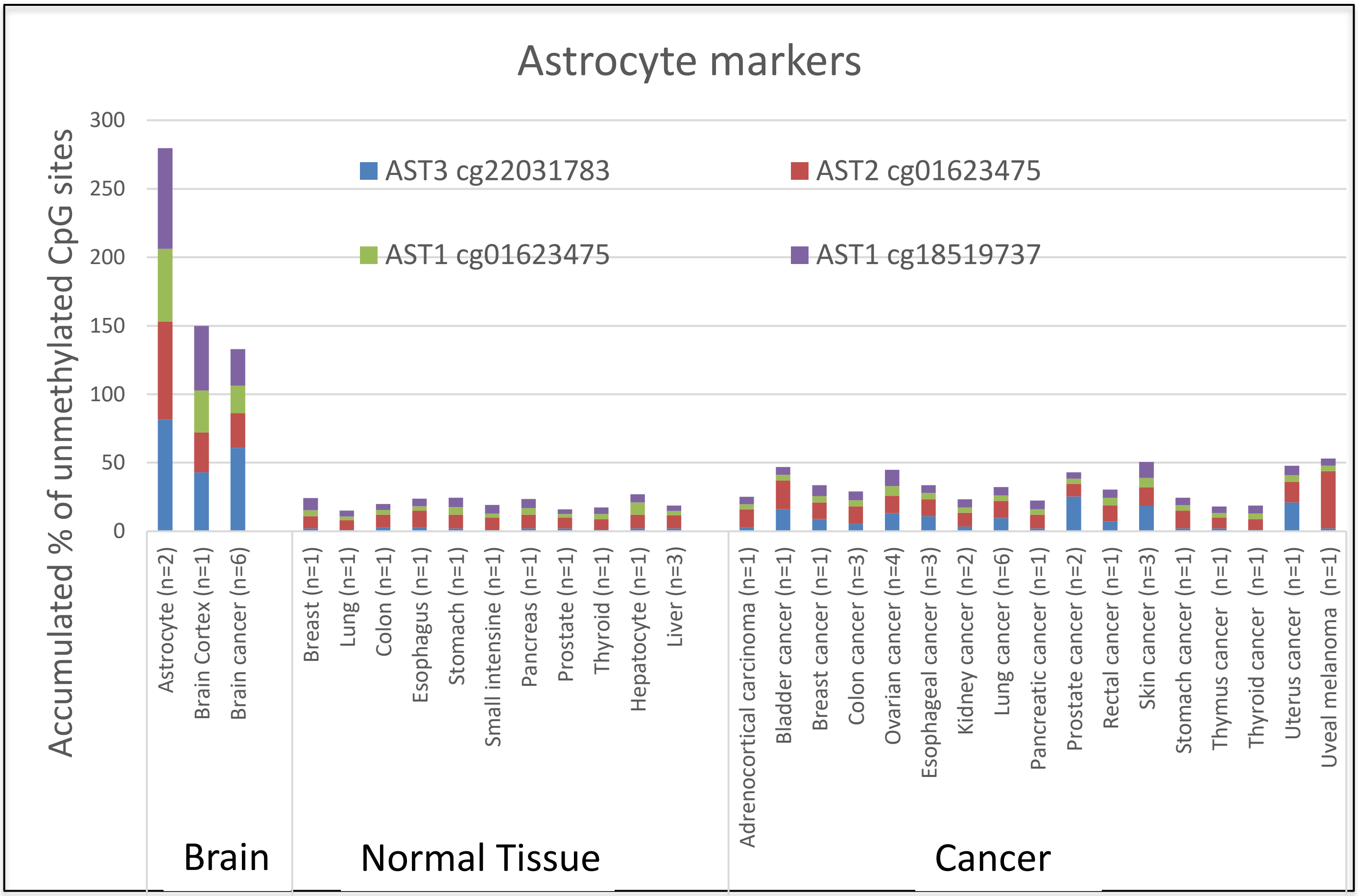
Astrocyte markers
AST3 cg22031783
AST2 cg01623475
AST1 cg01623475
AST1 cg18519737
Accumulated % of unmethylated CpG sites
300
250
200
150
100
50
0
Astrocyte (n=2)
Brain Cortex (n=1)
Brain cancer (n=6)
Breast (n=1)
Lung (n=1)
Colon (n=1)
Esophagus (n=1)
Stomach (n=1)
Small intensine (n=1)
Pancreas (n=1)
Prostate (n=1)
Thyroid (n=1)
Hepatocyte (n=1)
Liver (n=3)
Adrenocortical carcinoma (n=1)
Bladder cancer (n=1)
Breast cancer (n=1)
Colon cancer (n=3)
Ovarian cancer (n=4)
Esophageal cancer (n=3)
Kidney cancer (n=2)
Lung cancer (n=6)
Pancreatic cancer (n=1)
Prostate cancer (n=2)
Rectal cancer (n=1)
Skin cancer (n=3)
Stomach cancer (n=1)
Thymus cancer (n=1)
Thyroid cancer (n=1)
Uterus cancer (n=1)
Uveal melanoma (n=1)
Brain
Normal Tissue
Cancer

D

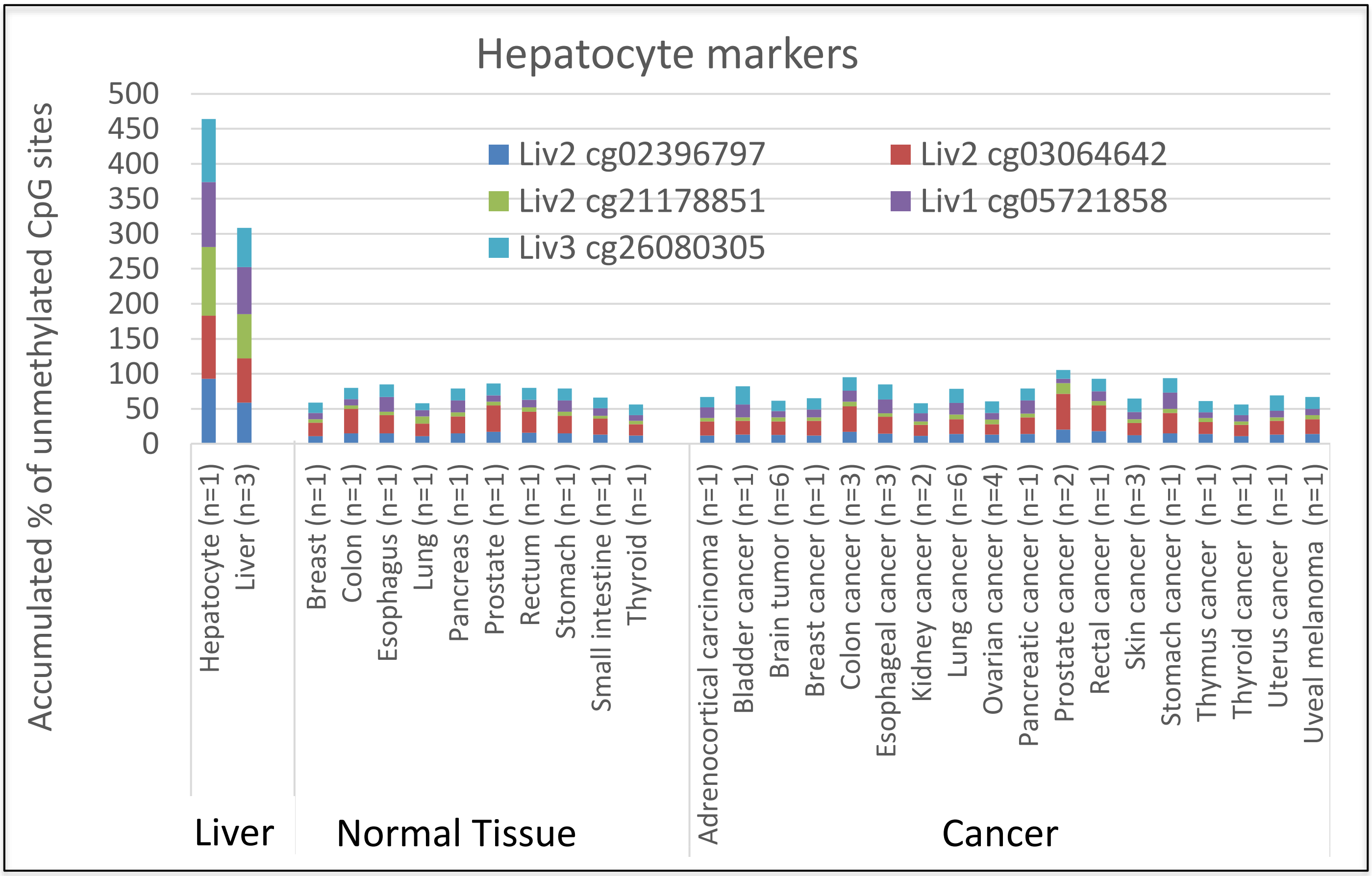
Hepatocyte markers
Liv2 cg02396797
Liv2 cg03064642
Liv2 cg21178851
Liv1 cg05721858
Liv3 cg26080305
Accumulated % of unmethylated CpG sites
500
450
400
350
300
250
200
150
100
50
0
Hepatocyte (n=1)
Liver (n=3)
Breast (n=1)
Colon (n=1)
Esophagus (n=1)
Lung (n=1)
Pancreas (n=1)
Prostate (n=1)
Rectum (n=1)
Stomach (n=1)
Small intestine (n=1)
Thyroid (n=1)
Adrenocortical carcinoma (n=1)
Bladder cancer (n=1)
Brain tumor (n=6)
Breast cancer (n=1)
Colon cancer (n=3)
Esophageal cancer (n=3)
Kidney cancer (n=2)
Lung cancer (n=6)
Ovarian cancer (n=4)
Pancreatic cancer (n=1)
Prostate cancer (n=2)
Rectal cancer (n=1)
Skin cancer (n=3)
Stomach cancer (n=1)
Thymus cancer (n=1)
Thyroid cancer (n=1)
Uterus cancer (n=1)
Uveal melanoma (n=1)
Liver
Normal Tissue
Cancer

Supplemental Figure S3: Methylation levels of CpG sites in our liver and brain-specific markers, using WGBS.(A) Neuron markers. (B) Oligodendrocyte markers. (C) Astrocyte markers. (D) Liver markers

A

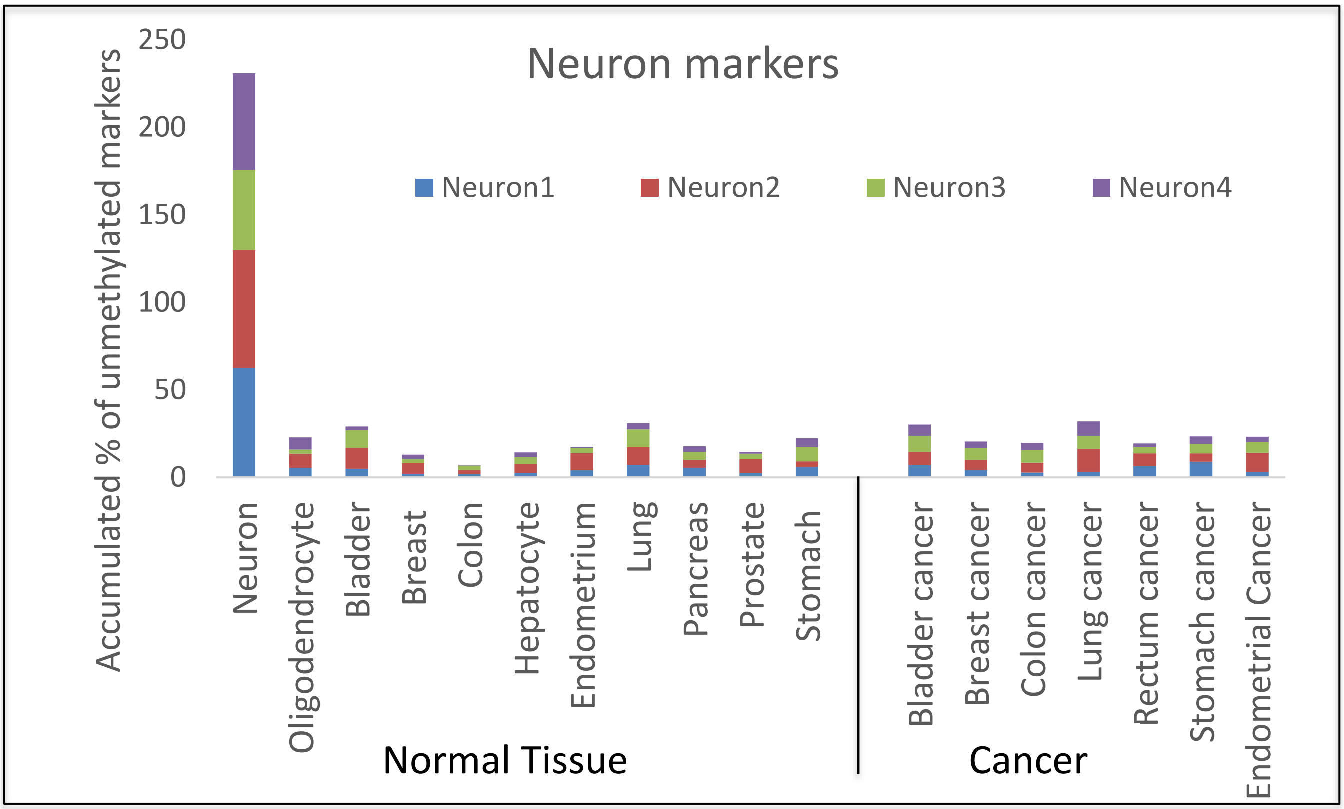

B

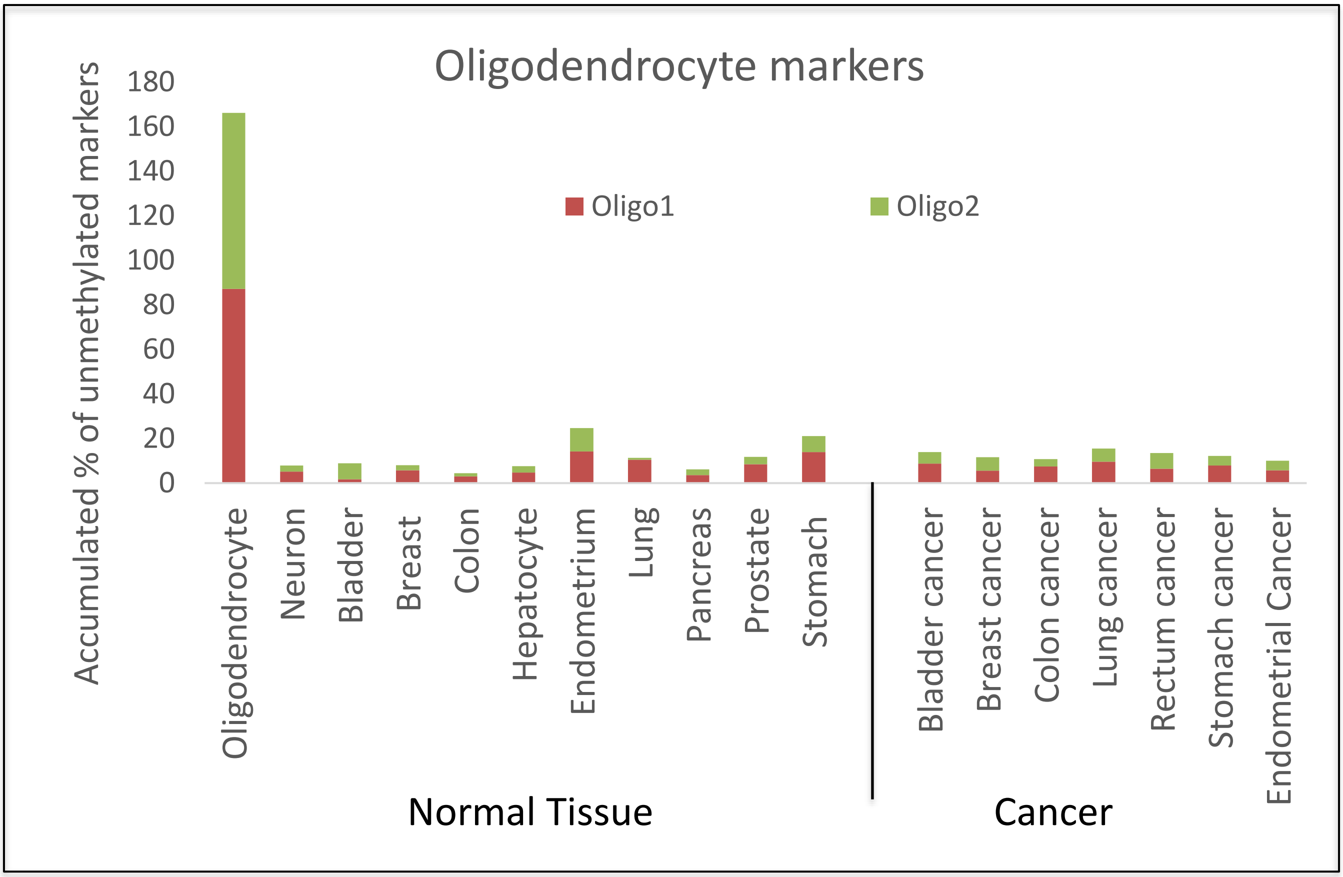

C

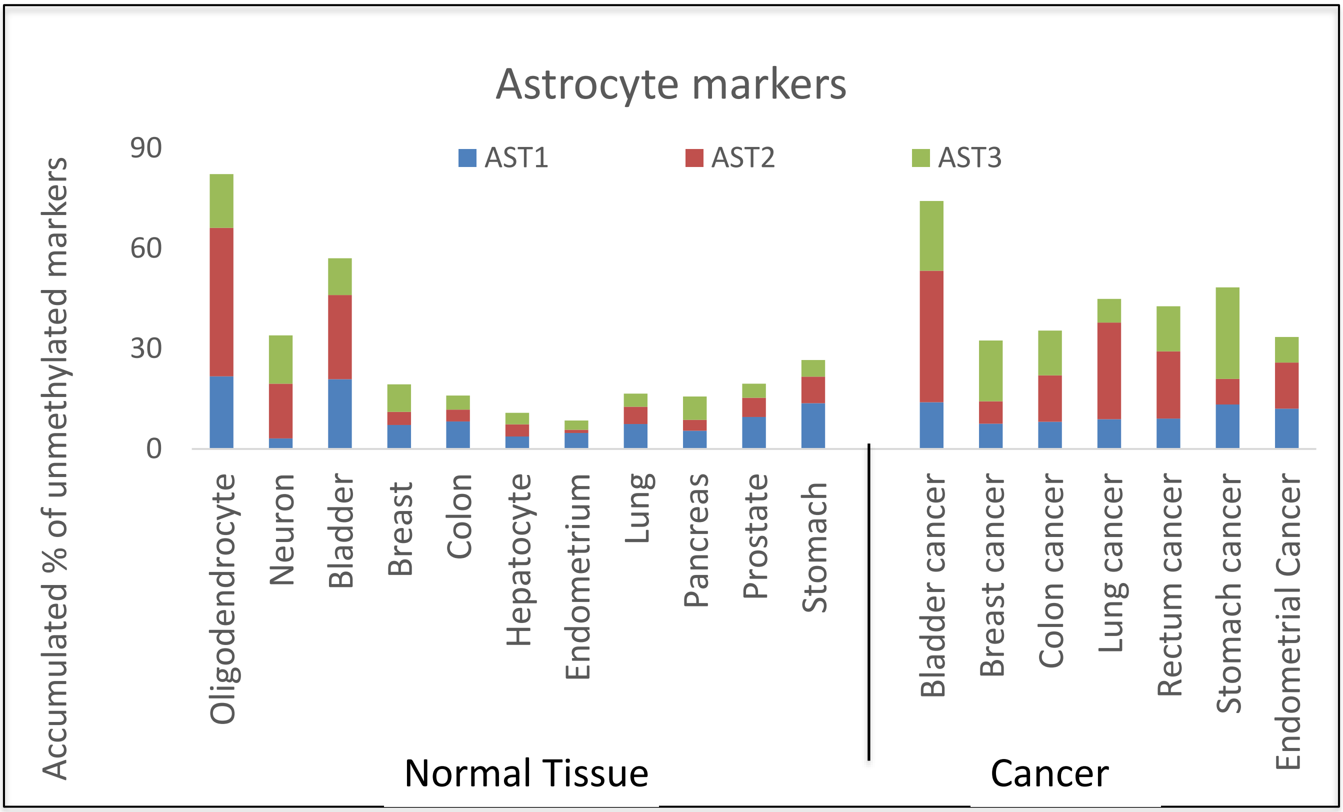

Astrocyte markers
AST1
AST2
AST3
Accumulated % of unmethylated markers
90
60
30
0
Oligodendrocyte
Neuron
Bladder
Breast
Colon
Hepatocyte
Endometrium
Lung
Pancreas
Prostate
Stomach
Bladder cancer
Breast cancer
Colon cancer
Lung cancer
Rectum cancer
Stomach cancer
Endometrial Cancer
Normal Tissue
Cancer

D

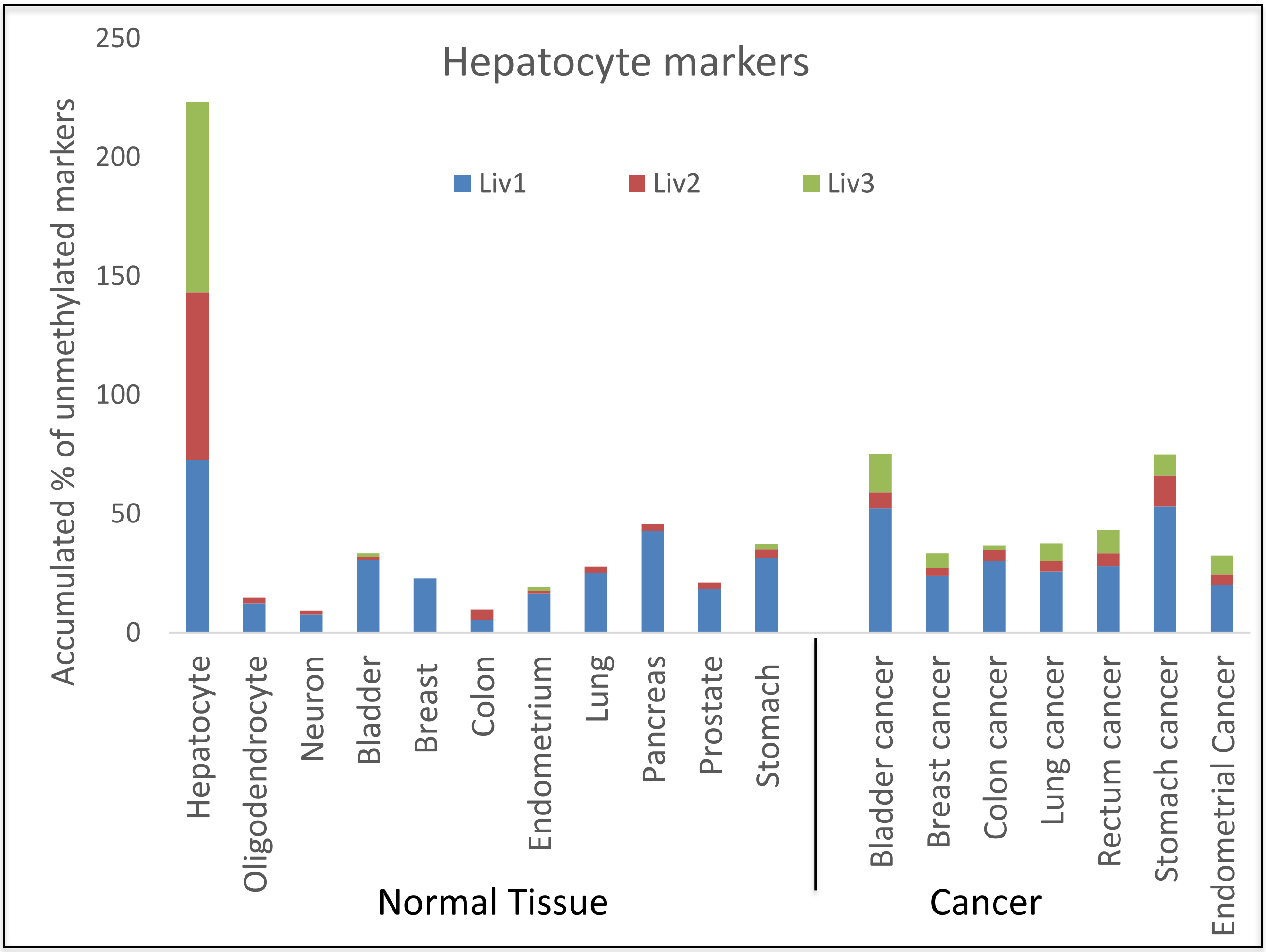

Hepatocyte markers
Liv1
Liv2
Liv3
Accumulated % of unmethylated markers
250
200
150
100
50
0
Hepatocyte
Oligodendrocyte
Neuron
Bladder
Breast
Colon
Endometrium
Lung
Pancreas
Prostate
Stomach
Bladder cancer
Breast cancer
Colon cancer
Lung cancer
Rectum cancer
Stomach cancer
Endometrial Cancer
Normal Tissue
Cancer
